# The Vicksburg Physicians

**Published:** 1879-02-20

**Authors:** C. J. Mitchell, E. T. Henry, J. R. Barnett, E. G. Banks, J. M. Hunt, T. G. Birchett, S. D. Robbins, W. T. Balfour, R. O’Leary


### *THE PHYSICIANS OF VICKSBURG.*

Through the kindness of a medical friend, we have a copy of the *Herald*, of Vicksburg, (date January 26), in which is published a beautiful and touching address by the physicians of the place relative to the trying ordeal through which they passed during the prevalence of the yellow fever. We regret that our space will not admit of its publication entire; but our readers can write for a copy, which, as it contains also a list of the deaths from the disease in nearly every place upon the river, will doubtless be valued by many.

The address portrays in eloquent terms the terrors of the epidemic, the noble and self-sacrificing work of the Howard Association, of the nurses, and the resident and visiting physicians, with appropriate allusion to the charities from neighboring towns and cities, the promptness and extent of which prevented the horrors of famine, which otherwise would have been superadded to those of the plague—"showing us in very deed to be one people, with one interest from sea to sea;" and in regard to the noble response for physicians and nurses from abroad, it is remarked: "That charitable persons with money are willing to give it in the cause of suffering humanity, is not astonishing; but that men and women can be found bold enough voluntarily to encounter the storm of death, that for two long months raged in every part of our city, is wonderful—its staggers belief. And we doubt if any other country on the wide earth can produce its parallel. In obedience to this call came about thirty doctors, and numerous nurses, many of them never having had yellow fever. Of these, Drs. Sappington, Barber, Norris, Blickfeldt, Roach, Happoldt, Blackman, Potts and Glass died. In our own county, Drs. Leatch, Nesmith, Birdsong and Monette were taken off." Reference is also made to the deaths "of Drs. Z. T. Woodruff, D. W. Booth, P. F. Whitehead and J. R. Hicks. Reverently and tenderly we record their names—ours to love and regret, but for a commonwealth to mourn."

The address concludes as follows: "To all our friends who have gone before us—let us hope to a better land—we would say most sincerely, we have missed you on the street, we have missed you at the bed-side, we have missed you everywhere.

> "And when the festive board is again spread,
> We will miss our loved dead,
> And will quaff in deepest silence,
> The fullest draft to their remembrance."

Signed by—

C. J. MITCHELL, M.D.
E. T. HENRY, M.D.
J. R. BARNETT, M.D.
E. G. BANKS, M.D.
J. M. HUNT, M.D.
T. G. BIRCHETT, M.D.
S. D. ROBBINS, M.D.
W. T. BALFOUR, M.D.
R. O'LEARY, M.D.

